# Significance of the mucinous component in the histopathological classification of colon cancer

**DOI:** 10.1007/s00595-015-1150-2

**Published:** 2015-03-21

**Authors:** Yuji Maeda, Sotaro Sadahiro, Toshiyuki Suzuki, Yasuo Haruki, Naoya Nakamura

**Affiliations:** 1 Departments of Surgery, Tokai University School of Medicine, 143 Shimokasuya, Isehara, Kanagawa 259-1193 Japan; 2 Department of Basic Medical Science, Tokai University, 143 Shimokasuya, Isehara, Kanagawa Japan; 3 Department of Pathology, Tokai University, 143 Shimokasuya, Isehara, Kanagawa Japan

**Keywords:** Colon cancer, Mucinous carcinoma, Mucinous component, Histological type

## Abstract

**Purpose:**

Mucinous carcinoma is often independently classified as a histological type of colon cancer, but there are currently no established diagnostic criteria. The relationship between the proportions of mucinous components to the oncological outcomes was examined to determine whether mucinous carcinoma should be classified as an independent histological type.

**Methods:**

The study group comprised 1,038 patients with colon cancer. The relationships between the survival rates and recurrence patterns with the mucinous component area ratio (MC area ratio) and clinical variables were evaluated.

**Results:**

Tumors were classified into three groups: Group 1 (MC area ratio, 0 %), Group 2 (1–49 %), and Group 3 (≥50 %). Of the 1038 tumors studied, 877 (84 %) were classified as Group 1, 123 (12 %) as Group 2, and 38 (4 %) as Group 3. The tumor size was significantly larger in Group 3, and an increased MC area ratio was significantly related to a higher proportion of right-sided tumors. Among patients with stage II or III disease, stage III disease, poorly differentiated adenocarcinoma, and no adjuvant chemotherapy were poor prognostic factors. There was no relationship between the MC area ratio and the survival or recurrence pattern.

**Conclusion:**

Mucinous carcinoma does not need to be classified as a separate histological type from ordinary differentiated adenocarcinoma.

## Introduction

The tumor node metastasis (TMN) classification (7th edition, American Joint Committee on Cancer) classifies colorectal adenocarcinoma into four histopathological grades: grade 1, well-differentiated adenocarcinoma; grade 2, moderately differentiated adenocarcinoma; grade 3, poorly differentiated adenocarcinoma, and grade 4, undifferentiated adenocarcinoma. There is no independent classification for colloid carcinoma or mucinous carcinoma. However, mucinous carcinoma or colloid carcinoma, and signet ring cell carcinoma are sometimes classified as independent histological entities [1–3].

There are currently no established definitions or diagnostic criteria for mucinous carcinoma. For example, a diagnosis of mucinous carcinoma required an MC area ratio of more than 60 % on largest cross sections of tumors in the studies by Symonds et al. and Umpleby et al. [4, 5]. However, mucinous carcinoma was defined as carcinoma with >50 % of tumor volume showing extracellular mucin in another study [6]. In the WHO classification, mucinous carcinoma is defined as an adenocarcinoma in which >50 % of the lesion is composed of pools of extracellular mucin [7]. Hogan et al. recently reported that mucinous adenocarcinoma defined according to the WHO criteria was associated with a reduced risk of death and better survival than non-mucinous adenocarcinoma [8]. However, the oncological outcomes reported were not consistent. Some studies reported that an MC area ratio of 10 % or higher was associated with poor survival [9]. Others reported that the outcomes of patients with mucinous tumors were similar to those of patients with other histological tumor types [10].

The criteria proposed in Japan classify the histological type on the basis of the most dominant histological appearance [11]. These criteria classify colorectal adenocarcinoma into well-differentiated adenocarcinoma, moderately differentiated adenocarcinoma, poorly differentiated adenocarcinoma, mucinous carcinoma, and signet ring cell carcinoma. With regard to the histological type, about 10 % of colon cancer patients had mucinous carcinoma. Signet ring cell carcinoma is exceedingly rare, its incidence was 1 % or lower in previous reports [2, 3, 12]. The grading of these lesions is determined by the content and appearance of the glandular structures. Therefore, the frequencies of low-grade tumors were 70–77 % in classical adenocarcinoma, 55–70 % in mucinous carcinoma, and 7–10 % in signet ring cell carcinoma [2, 3, 12].

In the present study, we examined the relationship between the proportion of extracellular mucin [mucinous components (MC)] in colon cancer tissue to the oncological outcomes to determine whether mucinous carcinoma should be classified as an independent histological type of colon cancer.

## Methods

The subjects were 1038 patients who underwent surgery for primary colon cancer at Tokai University Hospital between January 1991 and December 2005. Patients with appendiceal cancer were excluded. Data from the prospectively maintained database of the Departments of Surgery and Pathology of Tokai University Hospital were reviewed.

The following clinical variables were studied: age, sex, tumor site, TNM stage, the administration of postoperative adjuvant chemotherapy, and the survival rates. For histopathological evaluations, tissue specimens were stained with hematoxylin and eosin, and the predominant histological type, the presence or absence of MC on the largest cross sections of tumors, and the ratio of the area of MC to that of the entire tumor (MC area ratio) were evaluated. The MC area ratios were separately evaluated by two physicians (YM and SS), and the mean value was adopted. If the difference in estimated values was 20 % or greater, the two physicians reassessed the specimens to determine the consensus MC area ratio.

Because colon cancers in which MC accounts for more than 50 % of the cancer tissue are classified as mucinous carcinomas by the WHO, the tumors were classified into three groups according to the MC area ratio: Group 1 (MC area ratio, 0 %), Group 2 (1–49 %), and Group 3 (≥50 %).

The statistical analyses were performed with the Chi-square test and Fisher’s exact test. A multivariate analysis was performed using a Cox proportional hazards model. Cumulative survival rates were calculated by the Kaplan–Meier method and were compared with the use of log-rank tests.

This study was approved by the institutional review board of our university (14R-015).

## Results

The demographic characteristics of the patients are shown in Table 1. The cecum, ascending colon, and transverse colon were defined as the right side of the colon, and the descending colon, sigmoid colon, and the rectosigmoid colon were defined as the left side of the colon. Overall, 422 patients had right-sided colon cancer, and 616 had left-sided colon cancer. The histological type was well-differentiated adenocarcinoma or moderately differentiated adenocarcinoma in 93 % of the patients, poorly differentiated adenocarcinoma in 2 %, and mucinous carcinoma in 4 %.

**Table 1:** Patient demographics and clinical characteristics

Sex (male/female)	624/414
Age (years, mean ± SD)	65 ± 12
Site	
Right sided	422 (41 %)
Left sided	616 (59 %)
Histological type	
Well differentiated	557 (53 %)
Moderately differentiated	413 (40 %)
Poorly differentiated	26 (2 %)
Mucinous	38 (4 %)
Signet ring cell	4 (0.4 %)
Histological grade	
Grade 1	557 (53 %)
Grade 2	419 (40 %)
Grade 3	62 (6 %)
TNM stage	
I	105 (10 %)
II	404 (39 %)
III	312 (30 %)
IV	217 (21 %)
Adjuvant chemotherapy (patients with Stage II/III)	
Administered	397 (55 %)
Not administered	282 (39 %)
Unknown	37 (5 %)

Curative resection was performed in all 716 patients with stage II or III disease. Among these patients, 397 (55 %) received postoperative adjuvant chemotherapy with an oral fluoropyrimidine, intravenous 5-fluorouracil plus leucovorin or, oral UFT plus leucovorin. Postoperative adjuvant chemotherapy was not given to 282 patients (39 %), and the status of postoperative adjuvant chemotherapy was unknown in 37 patients (5 %).

The proportions of MC are shown in Table 2. MC was not found in 877 patients (84 %) and was present to some degree in the other 161 patients (16 %). There was no consistent trend in the distribution of the 161 patients according to the MC area ratio (data not shown). The mean age did not differ among the three groups classified according to the MC area ratio. The tumor size was significantly larger in Group 3 than in the other groups. The proportion of patients with right-sided colon cancer increased significantly in parallel with the increase in the MC area ratio. In Group 3, the proportion of patients with advanced-stage disease was significantly higher than that in Group 1, but did not differ from that in Group 2.–

**Table 2:** Patient and tumor characteristics according to MC area ratio

MC area ratio cases (%)	Group 1	Group 2	Group 3	Group 1 vs. Group 2	Group 2 vs. Group 3	Group 1 vs. Group 3
	0 %	1–49 %	≧50 %	*P* value	*P* value	*P* value
	877 (84)	123 (12)	38 (4)			
Age	64.4 ± 11.9	65.0 ± 13.6	64.3 ± 16.4	0.67	0.82	0.96
Size (cm)	5.0 ± 3.8	5.1 ± 2.2	7.2 ± 2.5	0.60	<0.0001	<0.0001
Site						
Right/left	333/544	62/61	27/11	0.01	<0.0001	0.04
Stage						
I	96	9	0	0.29	0.19	0.01
II	336	50	18			
III	272	33	7			
IV	173	31	13			

The results of a univariate analysis of the relationships between various factors and the survival rates in patients with stage II or III disease are shown in Table 3. Stage III disease, poorly differentiated adenocarcinoma, and no adjuvant chemotherapy were poor prognostic factors. There was no significant difference in the survival among patients with different histological grades.

**Table 3:**
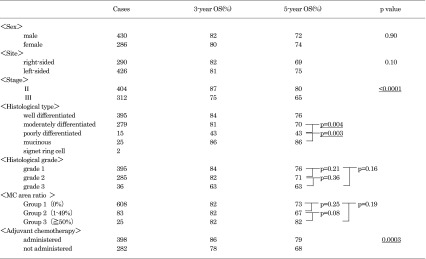
The results of a univariate analysis of factors associated with survival (patients with Stage II/III disease)

We also compared the survival rates in 716 patients according to the MC area ratios based on the following values: 10, 20, 30, and 50 %. There was no significant difference in the survival rates in any of the groups stratified by these values. The MC area ratio was not a significant determinant of the oncological outcomes.

The factors that were significantly related to survival or showed a trend toward being significantly related to survival were included in a multivariate logistic-regression model. The multivariate analysis showed that stage III disease, poorly differentiated adenocarcinoma, and postoperative adjuvant chemotherapy were significant prognostic factors (Table 4). The patterns of initial recurrence in patients with stage II or III disease are shown according to the MC area ratio in Table 5. There was no relationship between the MC area ratio and the recurrence pattern.

**Table 4:** The results of a multivariate analysis of factors associated with the overall survival (patients with Stage II/III disease)

Factor	HR	(95 % CI)	*P* value
Right sided (right sided vs. left sided)	0.80	(0.60–1.07)	0.13
Stage III (Stage III vs. Stage II)	1.88	(1.40–2.51)	0.001
Poorly differentiated(poorly vs. well/moderately, mucinous)	5.19	(1.55–17.3)	0.007
Adjuvant chemotherapy (administered vs. not administered)	0.57	(0.43–0.77)	0.001

**Table 5:** Patterns of recurrence according to the MC area ratio in patients with Stage II/III disease

MC area ratio	Group 1	Group 2	Group 3	Group 1 vs. Group 2	Group 2 vs. Group 3	Group 1 vs. Group 3
	0 %	1–49 %	≧50 %			
Patients (%)	609 (85)	83 (12)	25 (3)	*P* value	*P* value	*P* value
Liver	73	10	1	1.00	0.37	0.43
Lung	31	5	0	0.92	0.49	0.48
Peritoneum	30	6	1	0.53	1.00	0.91
Lymph node	19	3	2	1.00	0.44	0.71

## Discussion

Mucinous carcinoma is a histological subtype of colorectal cancer in which the MC component accounts for ≥50 % of the overall tumor volume [13]. Well-differentiated and moderately differentiated adenocarcinomas are the most common histological types of colorectal cancer. Mucinous carcinoma is relatively rare. The incidence of mucinous carcinoma was reported to be 10 % by Kang et al. 10 % by Hyngstrom et al. 11 % by Nitche et al. 15 % by Symonds et al. 11 % by Umpleby et al. and 6 % in Japan, suggesting that the incidence is lower in Japan than that in the Western countries [2–4, 12, 14–16]. However, this difference is at least partially attributable to the fact that the definition of, and diagnostic criteria for, mucinous carcinoma differs between Japan and Western countries.

Mucous areas were reported to be found in the tumor tissue in 30 or 31 % of patients with colorectal cancer. The intermingling of MC in tumor tissue is not rare in well-differentiated adenocarcinoma, moderately differentiated adenocarcinoma, or poorly differentiated adenocarcinoma [9, 10]. However, there are no established diagnostic criteria for mucinous carcinoma, and the criteria used in the previous studies have varied among investigators [1]. In our study, the rate of mucinous carcinoma was 4 %, which is generally consistent with that reported from other hospitals in Japan [17].

The histological grading was performed based on the content and appearance of the glandular structures. In the present study, signet ring cell carcinoma was excluded from the analysis, because the incidence of signet ring cell carcinoma was 1 % or less, and pure signet ring cell carcinoma is extremely rare. However, Sung et al. reported that 17 % of mucinous carcinomas included some extent of signet ring cells in the tumor tissue [6].

Some studies have reported that the incidence of mucinous carcinoma is higher in younger patients, whereas others have found no age-related difference [2, 5, 15, 18–20]. Consistent results have yet to be obtained. In our series, there was no relationship between age and the MC area ratio.

Compared with well-differentiated and moderately differentiated adenocarcinomas, mucinous carcinoma has been reported to develop more often in the right side of the colon [2, 4, 10, 15, 16, 21, 22]. In our series, an increase in the MC area ratio was associated with a higher proportion of right-sided tumors (**p < 0.0001).

Mucinous carcinoma has been reported to be associated with a large tumor diameter (15). In our study, an MC area ratio of 50 % or higher was associated with a significantly larger tumor size than an MC area ratio of less than 50 %. With regard to the sites of recurrence, mucinous carcinoma has been reported to have a high incidence of peritoneal metastasis and a low incidence of liver metastasis [14, 15, 21, 23, 24]. In our series, however, there was no relationship between the MC area ratio and recurrence pattern.

A multivariate analysis revealed that postoperative adjuvant chemotherapy was a significant prognostic factor. The indications for adjuvant chemotherapy and the treatment regimens were decided by the attending physicians at the participating hospitals during the study period. The presence of a mucinous component was not a criterion for selecting adjuvant chemotherapy.

In patients with stage II or III colon cancer who underwent curative resection, a histological type of poorly differentiated adenocarcinoma was a poor prognostic factor. Consistent results have not been obtained for the outcomes of mucinous carcinoma, with some studies reporting that an MC area ratio of 10 % or higher was associated with poor survival, and another reporting that the outcomes were better, and others reporting that outcomes were similar to those of other histological types [8–10]. Kang et al. analyzed data from a database of the Surveillance, Epidemiology, and End Results Program of the National Cancer Institute from 1991 through 2000 and found that the outcomes of mucinous carcinoma were similar to those of adenocarcinoma [2]. In our study, the three-year survival rates of patients with well-differentiated and moderately differentiated adenocarcinomas were 84 and 81 %, respectively, similar to that, i.e., 82 % in Group 3 with an MC area ratio of 50 % or higher. Umpleby et al. classified patients into a high mucinous group, with an MC area ratio of higher than 80 %, and a moderate mucinous group, with an MC area ratio of 60–80 % [14]. The outcomes were poorer in the high mucinous group in that study. In the present study, we compared the survival rates according to the MC area ratios at the borderline values of 10, 20, 30, and 50 %. However, there was no difference in the survival rates in any of these values.

A small number of patients have marked mucinous components in cancer tissue, although this histological feature was not associated with the oncological outcomes in the present study. It might be best to record this histological feature as another histological subtype, rather than a completely separate histological type.

## Conclusions

In patients with adenocarcinoma of the colon, poorly differentiated adenocarcinoma is a significant predictor of poor outcomes, whereas the MC area ratio is not a significant prognostic factor. Our results suggest that mucinous carcinoma histologically characterized by a preponderance of MC does not need to be classified as a separate histological type from ordinary differentiated adenocarcinoma.
